# Paradoxical Increase in TAG and DAG Content Parallel the Insulin Sensitizing Effect of Unilateral DGAT1 Overexpression in Rat Skeletal Muscle

**DOI:** 10.1371/journal.pone.0014503

**Published:** 2011-01-14

**Authors:** Silvie Timmers, Johan de Vogel-van den Bosch, Matthijs K. C. Hesselink, Denis van Beurden, Gert Schaart, Maria Joao Ferraz, Mario Losen, Pilar Martinez-Martinez, Marc H. De Baets, Johannes M. F. G. Aerts, Patrick Schrauwen

**Affiliations:** 1 Top Institute Food and Nutrition (TIFN), Wageningen, The Netherlands; 2 Department of Human Biology, Maastricht University, Maastricht, The Netherlands; 3 Human Movement Sciences, School for Nutrition, Toxicology and Metabolism (NUTRIM), Maastricht University, Maastricht, The Netherlands; 4 Department of Medical Biochemistry, Academic Medical Center, University of Amsterdam, Amsterdam, The Netherlands; 5 Department of Neuroscience, School of Mental Health and Neuroscience, Maastricht University, Maastricht, The Netherlands; University of Padova, Italy

## Abstract

**Background:**

The involvement of muscle triacylglycerol (TAG) storage in the onset of insulin resistance is questioned and the attention has shifted towards inhibition of insulin signalling by the lipid intermediate diacylglycerol (DAG). The enzyme 1,2-acylCoA:diacylglyceroltransferase-1 (DGAT1) esterifies a fatty acyl-CoA on DAG to form TAG. Therefore, the aim of the present study was to investigate if unilateral overexpression of DGAT1 in adult rat Tibialis anterior (TA) muscle will increase conversion of the lipid intermediate DAG into TAG, thereby improving muscle insulin sensitivity.

**Methodology/Principal Findings:**

The DGAT1 gene construct was injected in the left TA muscle of male rats on chow or high-fat (45% kcal) diet for three weeks, followed by application of one 800 V/cm and four 80 V/cm pulses, using the contralateral leg as sham-electroporated control. Seven days after electroporation, muscle specific insulin sensitivity was assessed with a hyperinsulinemic euglycemic clamp using 2-deoxy-[3H]glucose. Here, we provide evidence that unilateral overexpression of DGAT1 in TA muscle of male rats is associated with an increased rather than decreased DAG content. Strikingly, this increase in DAG content was accompanied by improved muscle insulin sensitivity. Interestingly, markers of muscle lipolysis and mitochondrial function were also increased in DGAT1 overexpressing muscle.

**Conclusions/Significance:**

We conclude that unilateral DGAT1 overexpression can rescue insulin sensitivity, possibly by increasing DAG and TAG turnover in skeletal muscle. In case of a proper balance between the supply and oxidation of fatty acids in skeletal muscle, the lipid intermediate DAG may not exert harmful effects on insulin signalling.

## Introduction

Obesity is characterized by excessive fatty acid mobilization, giving rise to many obesity-related metabolic complications including insulin resistance [1], [2]. Indeed, a high rate of fatty acid availability and subsequent uptake by skeletal muscle can augment intramuscular lipid deposition [1], [2], and there is a strong correlation between the intramyocellular lipid (IMCL) concentration and the severity of insulin resistance, both in humans and rodents [3], [4], [5], [6]. However, evidence from the last decade supports the hypothesis that IMCL accumulation does not directly affect insulin action [7], [8], [9], [10], rather other fatty acid intermediates, such as DAG, are proposed to impede insulin signalling [11], [12], [13]. In fact, endurance trained athletes are considered among the most insulin-sensitive people, despite having high concentrations of IMCL; a finding that is referred to as “the athlete's paradox” [7], [8], [9], [10]. Therefore, IMCL is considered a reservoir for fatty acids that can be used for substrate delivery, such as during exercise, and if IMCL accumulation is accompanied by high fat oxidative capacity, detrimental effects of IMCL may be limited. This also implies that under conditions of high fatty acid flux, as with high-fat diets and obesity, limiting the accumulation of detrimental fatty acid metabolites such as DAG by improving the partitioning of fatty acids towards storage as IMCL, is likely to blunt lipid related insulin resistance [9].

DGAT1 is a key enzyme that catalyzes the final step in triglyceride synthesis. Overexpressing this enzyme results in increased triglyceride synthesis, increased fatty acid oxidation and preservation of insulin sensitivity despite the increased amount of fatty acids offered to the muscle [14], [15]. It has therefore been suggested that elevated DGAT1 activity could be an explanation for the athlete's paradox. Indeed, Schenk and Horowitz [16] showed that one acute bout of exercise leads to an increase in DGAT1 expression and improved insulin sensitivity. Also in rodents, exercise increases myocytic DGAT1 activity, and transgenic overexpression of DGAT1 in skeletal muscle in mice is sufficient to replicate the exercise paradox characterized by increased muscle lipid content coupled with increased muscle insulin sensitivity [14], [17]. However, life-long muscle overexpression of DGAT1 may not resemble the situation that is observed with endurance training, and may in fact lead to phenotypic compensatory changes, such as a lower body weight that was observed in mice overexpressing DGAT1 [14]. Therefore, we here aimed to examine the impact of acute unilateral, muscle-specific overexpression of DGAT1 in adult rats on skeletal muscle lipid metabolism. To this end, we transiently overexpressed DGAT1 by using gene electroporation in left TA muscle of adult rats while using the right leg of the same animal as a sham-electroporated control [18]. We hypothesized that overexpression of DGAT1 in TA muscle of adult male Wistar rats would lead to an increase in IMCL content and a decrease in DAG content, leading to improved insulin sensitivity. Strikingly, we observed that DGAT1 overexpression was paralleled by an increased rather than decreased DAG content yet improved insulin sensitivity. Interestingly, key proteins in myocellular lipolysis and oxidative substrate metabolism were increased upon DGAT1 overexpression.

## Methods

### Ethics statement

The experimental protocol was approved by the Institutional Animal Care and Use Committee of Maastricht University and complied with the principles of laboratory animal care (approval number 2006-024). During the experiments all efforts were made to minimize suffering of the animals.

### Animals and diets

Twenty-two, eight-week old male Wistar rats were obtained from Charles River Laboratories and were housed individually in an environmentally controlled laboratory (temperature 22±1°C and relative humidity 55±2%). Rats were randomly divided in two groups and were allowed ad libitum access to food and tap water. One group (n = 10) received standard CHOW (10 energy % as fat) (Ssniff, Germany) and the other group (n = 12) received a high-fat diet (HFD) that contained 45 energy % of fat derived from lard (Research Diets, D01060502, Wijk bij Duurstede, The Netherlands). Diets were provided for three weeks.

### Cannulation

Two weeks before the end of the experiment the rats were sedated under isoflurane anaesthesia and permanent indwelling catheters were placed in the right jugular vein (for the sampling of blood) and the left carotid artery (for the infusion of insulin and glucose) under aseptic conditions. Both catheters were tunnelled subcutaneously and exteriorized at the top of the head, where they were attached. To maintain patency, the catheters were filled with a mixture of 85% glycerol (Merck, Darmstadt, Germany) and 500 U/ml heparin (Leo Pharma BV, Breda, The Netherlands). All rats regained pre-surgery body mass within 3 days.

### Electroporation

One-week before the hyperinsulinemic euglycemic clamp overexpression of mouse DGAT1 in the left Tibialis anterior (TA) muscle of the rat was obtained, with the right TA as sham-electroporated internal control. DNA electroporation was done under isoflurane anaesthesia. Left TA muscles were transcutaneously injected with 150 µg (2 µg/µl) pcDNA sport DGAT1-construct (pCMV-sport6, Invitrogen, Breda, The Netherlands) in 0.9% sterile NaCl. Right TA muscles were injected with 150 µg (2 µg/µl) empty vector. Within 15 seconds after the last injection 5 electric pulses were applied by two stainless steel plate electrodes placed at each side of the leg to obtain transient permeabilization of the cell membrane, facilitating the passage of the DNA. One high voltage pulse of 800 V/cm and four low voltage pulses of 80 V/cm at 1 Hz were generated by an ECM 830 electroporator (BTX, San Diego) as described before [19], [20], [21]. Shaving the leg and the application of conductive gel minimized impedance.

### Hyperinsulinemic euglycemic clamp

Hyperinsulinemic euglycemic clamping of conscious rats was performed after a 6 h fast. A primed continuous infusion of insulin (Actrapid HM; Novo Nordisk, Copenhagen, Denmark) was administered at a rate of 13 mU/kg/min for 120 min. Blood glucose values were monitored at 10-min intervals throughout the clamp. The glucose infusion rate (GIR) was adjusted to maintain blood glucose concentration within the range of 4.5–5.5 mmol/L. Insulin stimulated glucose disposal *in vivo* within the individual TA muscles was studied according to a previously described method [22]. Upon completion of the clamp, the rats were killed with an overdose of sodium pentobarbital and TA muscles were quickly dissected and frozen in liquid nitrogen-cooled isopentane and stored at −80°C until further analyses.

### Deoxy glucose measurement

Tibialis anterior muscles were cut in three parts and the middle part was ground with a N_2_-cooled mortar and pestle. After homogenisation, the samples were heated to 99°C for 30 min, cooled and centrifuged for 5 min at 13,000 rpm. 2-deoxy-[1-3H]-D-glucose-phosphate was separated from 2DG in the supernatant by binding to a Dowex column (Dowex 1*8-100, ion exchange resin, Sigma). Bound 2-deoxy-[1-3H]-D-glucose-phosphate was eluted from the column by using the eluate buffer (formic acid, ammoniumacetate in mQ, pH 4.9). 14 ml LCD scintillation fluid was added to 1 ml eluate, and this cocktail was counted in the scintillation counter for 5 min.

### Histological analysis of intramyocellular lipids (IMCL) and quantification of DGAT1

Cryosections (5 µm) from the midbelly region of the TA muscle were stained for neutral lipids with Oil Red O and DGAT1 overexpresssion was quantified as previously described [15].

### Electron microscopy

In a subset of 4 CHOW and 4 high-fat fed mice both the DGAT1 overexpressing muscle as well as the empty-vector control condition were processed for routine transmission electron microscopy. In brief, small blocks of muscle tissue (2*2*2 mm) from both legs were fixed by glutaraldehyde and post-fixed by osmiumtetroxide and epon embedded. Semi-thin sections (4 microns) were cut to confirm longitudinal fiber orientation by light microscopy. Subsequently, ultrathin (80–90 nm) sections were cut and stained and contrasted with uranyl acetate and lead-citrate. Then, sections were examined in a Philips CM100 transmission electron microscope (Philips, Eindhoven, The Netherlands). Per muscle 15–20 images were grabbed and analyzed by an experienced electron microscopist blinded for the conditions. Using the NIH image analysis program all individual lipid droplets were identified, counted and encircled to compute lipid droplet area and derived morphometric parameters. Frequency distributions were computed and plotted using Excel for Windows.

### DAG measurement

Total lipids were extracted from frozen muscle using the method of Folch et al. [23]. The extracts were filtered and lipids recovered in the chloroform phase. DAG was isolated using thin-layer chromatography on Silica Gel 60 A plates developed in petroleumbenzin-diethyl ether-acetic acid (120∶25∶1.5 by volume) and visualized by rhodamine 6G. The DAG band was scraped from the plate and methylated using a mixture of toluene-methanol- (BF3/methanol 14%) (20%-55%-25% by volume), as described by Morrison and Smith [24]. The methylated fatty acids were extracted with hexane and analyzed by capillary gas liquid chromatography using a 50 m ×0.25 mm CP-sil 88 silica column (varian) with helium as carrier gas at a flow of 130 kPa. The column oven was maintained at 165°C for 10 min and increased at a rate of 5°C/min to 190°C. This temperature was maintained for 15 min. Then temperature was increased to 230°C for 22 min with a flow rate of 2°C/min. Fatty acid methyl esters were identified by comparing retention times to those of known standards. Inclusion of an internal standard, 19∶0 (dinonadecanoic acid), and an average molecular mass for each fatty acid methyl ester permits quantification of the amount of DAG in the sample, expressed as micromolar per gram of wet tissue weight.

### Ceramide measurement

Ceramide content was determined as previously described (25) with slight modifications. Briefly, 50 µl of muscle homogenate was extracted with 600 µl of CHCl_3_/MeOH 1/2 (vol/vol). The extract was centrifuged for 10 min at 14,000 *g* and the pellet discarded. 500 µl of CHCl_3_/MQ-H_2_O 1/1.5 (vol/vol) was added, mixed, and centrifuged for 3 min at 14,000 *g* to separate the phases. The lower phase was collected and the upper phase re-extracted with 400 µl of CHCl_3_. The combined lower phases were dried under N_2_ flow, taken up in 500 µl of freshly prepared 0.1 M NaOH in MeOH, and deacetylated in a microwave (SAM-155, CEM corp.) for 60 min. 50 µl of this solution was derivatized with 25 µl *o*-phtaldehyde (OPA) reagent. The OPA-derivatized lipids were separated by HPLC and ceramides were quantified as previously described, using C17 sphinganine as an internal standard [25].

### Western blotting

Muscle samples were homogenized as described previously [26] and processed for standard SDS-PAGE and Western blotting. Protein concentration was assessed, and equal amounts of protein were loaded per lane. Actin was used as a loading control. Membranes were incubated with antibodies against OXPAT (GP31, Progen, Heidelberg, Germany), OXPHOS (MS601; MitoSciences, Eugene, OR, USA), PGC1α (516557; Calbiochem; VWR International BV, Amsterdam, The Netherlands) and mUCP3 [27], ADRP (GP40, Progen), ATGL (2138; Cell Signalling Technology; Bioké; Leiden, The Netherlands) and CGI58 (NB110-41576; Novus Biologicals, Littleton, CO, USA). Blots incubated with OXPAT, OXPHOS, PGC1α and mUCP3 were probed with IRDye800-conjugated or IRDye700-conjugated secondary antibodies (Rockland, Gilbertsville, PA, USA and LICOR Biosciences, Westburg, Leusden, The Netherlands), and bands at a molecular weight corresponding to the control samples were quantified using the Odyssey infrared imaging system (LICOR Biosciences, Wateringen, the Netherlands). Blots detecting ADRP, ATGL and CGI58 were probed with the appropriate horse radish-conjugated antibodies and after incubation specific protein bands were visualized by chemiluminescence and analyzed by Chemidoc XRS system (Bio-Rad, Veenendaal, The Netherlands).

### Statistics

Results are presented as means ± SEM. Differences between legs within one rat were analysed by paired samples t-test. Between groups, differences were analysed with an independent t-test. The accepted level of statistical significance was p<0.05 for all analyses. Al calculations were done using the Statistical Package for the Social Sciences (SPSS 16.0 software).

## Results

### Body mass

Body mass gradually increased over the three weeks of dietary intervention in both the CHOW- and HFD-fed rats. However, this short-term dietary intervention did not result in differences in body mass between diets (300±10 g and 290±10 g in HFD- vs. CHOW-fed rats, p = 0.70).

### DGAT1 overexpression, intramyocellular lipid levels

**Figure 1:**
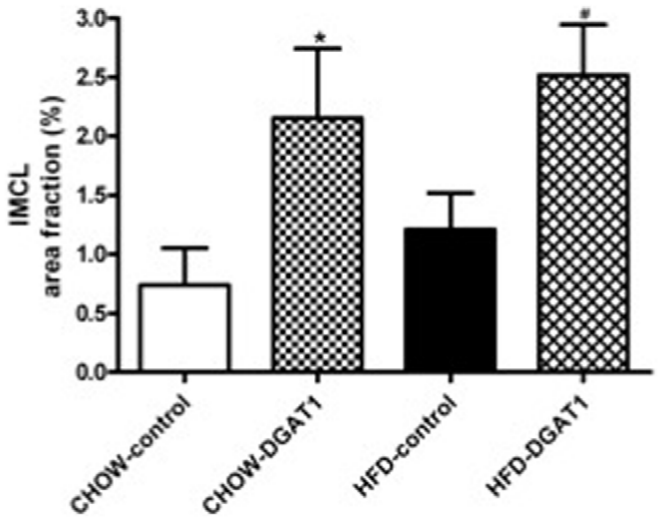
Electroporation of DGAT1 in TA muscle of rats on CHOW and HFD is associated with an increased partitioning of fatty acids towards IMCL. IMCL levels after three weeks of dietary intervention, area fraction reflects the percentage of the total measured cell surface covered by lipid droplets. Data are expressed as mean ± SEM (n = 10–12). *P<0.05 CHOW-DGAT1 vs. CHOW-control, **^#^** P<0.05 HFD-DGAT1 vs. HFD-control.

One week after electroporation, immunohistochemistry revealed a widely distributed DGAT1 overexpression in the muscle fibres of left TA muscle of rats; both in rats receiving the HFD as well as the CHOW. The mean green intensity of images from the DGAT1 electroporated leg was significantly higher than observed in the sham-electroporated contralateral control leg (25.77±2.09 AU vs. 8.32±1.68 AU in HFD-DGAT1 vs. HFD-control, p<0.001, and 25.73±3.17 AU vs. 7.37±1.79 AU in CHOW-DGAT1 vs. CHOW-control, p<0.001), indicating significant overexpression of DGAT1. The DGAT1 overexpression was associated with a ∼52% increase in intramyocellular lipids in the HFD-DGAT1 overexpressing leg and a ∼66% increase in muscle triglyceride content in the CHOW-DGAT1 overexpressing leg, compared to control legs of the animals (p = 0.018, HFD-DGAT1 vs. HFD-control leg, and p = 0.044, CHOW-DGAT1 vs. CHOW-control leg, **figure 1**).

### Diacylglycerol content

**Figure 2:**
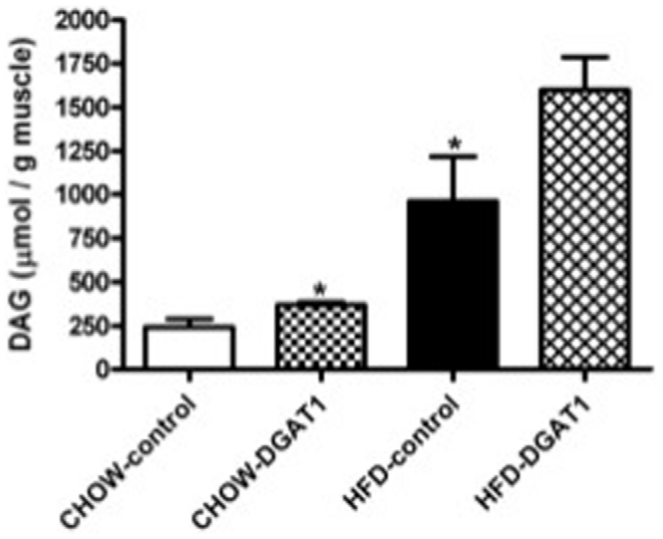
Three weeks of high-fat feeding is associated with an increased TA DAG content compared to rats on CHOW. DGAT1 overexpression leads to an increase in TA DAG content in rats on CHOW and tended to increase DAG content in rats on HFD. Data are expressed as mean ± SEM (n = 6). *P<0.05 CHOW-DGAT1 and HFD-control vs. CHOW-control.

To determine whether the increased IMCL content in TA muscle overexpressing DGAT1 was accompanied by a reduction of DAG, we measured the latter in the legs overexpressing DGAT1 as well as in their empty vector control legs (**figure 2**). Three weeks of high-fat feeding was associated with a ∼75% increase in DAG content (p = 0.039). Remarkably, overexpressing DGAT1 led to a ∼30% increase in DAG content in the HFD-DGAT1 overexpressing leg and a ∼35% increase in DAG content in CHOW-DGAT1 overexpressing leg, compared to control legs of the animals (p = 0.073, HFD-DGAT1 vs. HFD-control leg, and p = 0.045, CHOW-DGAT1 vs. CHOW-control leg).

### Ceramide content

**Figure 3:**
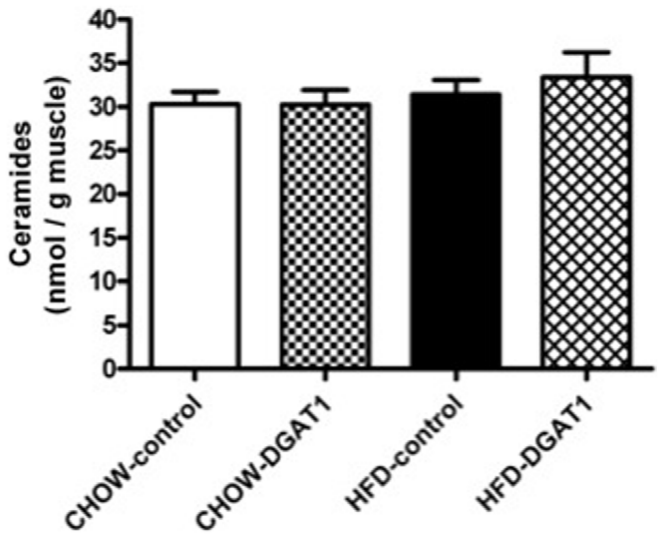
Three weeks of high-fat feeding is not associated with an increased TA ceramide content compared to rats on CHOW. DGAT1 overexpression does not result in an increase in TA ceramide content in rats on CHOW or HFD. Data are expressed as mean ± SEM (n = 10–12).

Ceramide content was measured in TA muscle overexpressing DGAT1 and in the contralateral empty vector electroporated legs to determine whether the increase in IMCL content is linked to changes in muscle ceramide levels (**figure 3**). Compared the CHOW-fed animals, three weeks of high-fat feeding did not increase ceramide content in TA muscle. Furthermore, also DGAT1 overpression did not affect ceramide levels both in CHOW-DGAT1 overexpressing legs and in HFD-DGAT1 overexpressing legs compared to their controls, respectively.

### Insulin sensitivity

**Figure 4:**
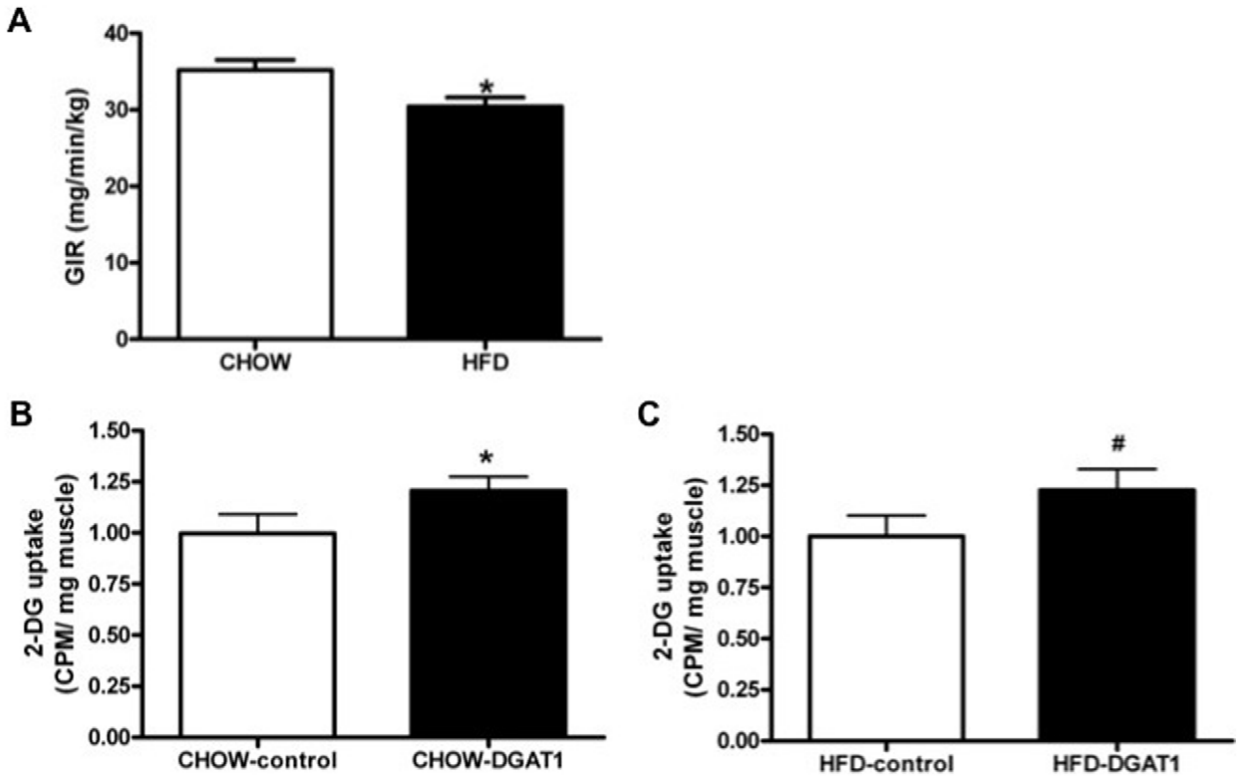
Three weeks of high-fat feeding compared to rats on CHOW decreased whole-body insulin sensitivity as assessed by hyperinsulinemic eulgycemic clamp. DGAT1 overexpression in TA muscle increased muscle specific insulin sensitivity both in rats on CHOW as HFD. (**A**) Steady-state whole-body glucose infusion rates in rats on CHOW and HFD for three weeks. (**B**) 2DG uptake in TA muscle of CHOW fed rats after bolus injection of 2-deoxy-[3H]glucose under the same clamping conditions as in A. (**C**) 2DG uptake in TA muscle of HFD fed rats after bolus injection of 2-deoxy-[3H]glucose under the same clamping conditions as in A. Data are expressed as mean ± SEM (n = 10–12). *P<0.05 CHOW-DGAT1 and HFD-control vs. CHOW-control, **^#^** P<0.05 HFD-DGAT1 vs. HFD-control.

One week after electroporation of DGAT1 whole-body and tissue insulin sensitivity were assessed *in vivo* by hyperinsulinemic-euglycemic clamps in combination with radioisotope-labelled infusion of 2-deoxy-[3H]glucose (2DG). Compared to CHOW-fed animals, three weeks of high-fat feeding induced whole body insulin resistance as reflected by a significantly lower glucose infusion rate (30.4±1.2 mg/min/kg vs. 35.2±1.3 mg/kg/min in HFD- vs. CHOW-fed animals, p = 0.02, **figure 4A**). In CHOW fed rats, 2DG uptake was significantly elevated in DGAT1 overexpressing TA muscle compared to their empty vector control legs (p = 0.036, **figure 4B**). Also upon high-fat feeding, DGAT1 overexpression significantly increased 2DG uptake in TA muscle compared to the empty vector control legs of these rats (p = 0.05, **figure 4C**). These results were not confounded by altered basal GLUT4 expression, as this was not affected by DGAT1 overexpression (data not shown).

### Markers of mitochondrial function and biogenesis (OXPHOS, PGC1α, UCP3)

**Figure 5:**
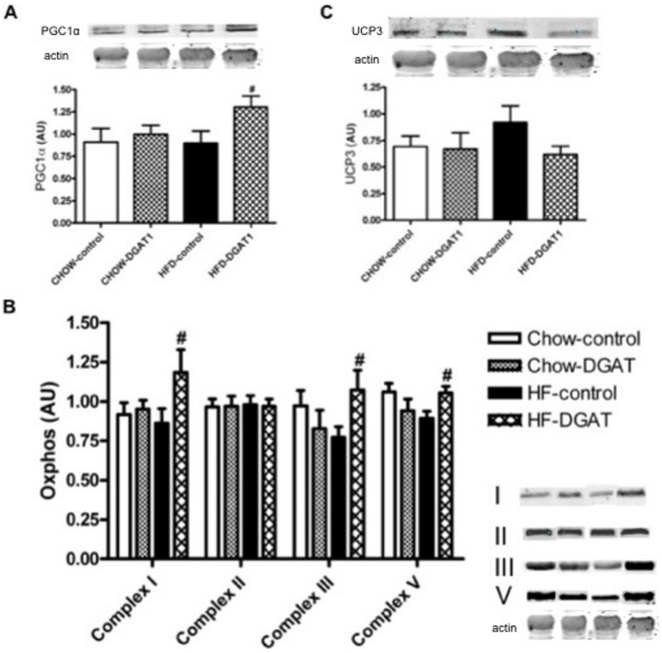
DGAT1 overexpression is associated with an increased mitochondrial function and biogenesis in HFD-fed rats. (**A**) Western blot analysis of PGC1α (**B**) OXPHOS complexes and (**C**) UCP3 in rat TA muscle. Data are expressed as mean ± SEM (n = 10–12). **^#^** P<0.05 HFD-DGAT1 vs. HFD-control.

To test if the increase in lipid content in DGAT1 overexpressing muscle was accompanied by a compensatory increase in mitochondrial capacity, we determined protein levels of major mitochondrial markers. DGAT1 overexpression was associated with a ∼32% increase in PGC1α in the HFD-DGAT1 overexpressing leg compared to the control leg of the animals (p = 0.04, **figure 5A**), suggesting an increased mitochondrial biogenesis. High fat feeding did not affect the PGC1α content in TA muscle. DGAT1 overexpression in CHOW animals had no significant effect on PGC1α expression (p = 0.64, **figure 5A**). Western blotting of the OXPHOS complexes revealed that DGAT1 overexpression, in rats fed a HFD, led to an increase in protein levels of the individual complexes I, III and V, suggesting an increased mitochondrial density (Complex I: 1.2±0.4 AU vs. 0.86±0.26 AU, p = 0.007, Complex II: 0.97±0.31 AU vs. 0.98±0.31 AU, p = 0.89 Complex III: 1.0±0.32 AU vs. 0.77±0.23 AU, p = 0.0023, Complex V: 1.0±0.30 AU vs. 0.89±0.26 AU, p = 0.014 in HFD-DGAT1 vs. HFD-control leg, **figure 5B**). DGAT1 overexpression did not enhance mitochondrial density in rats receiving CHOW represented as differences in protein expression of the individual complexes (Complex I: 0.95±0.39 AU vs. 0.92±0.37 AU, p = 0.72, Complex II: 0.97±0.31 AU vs. 0.96±0.31 AU, p = 0.96, Complex III: 0.83±0.29 AU vs. 0.97±0.34 AU, p = 0.35, Complex V: 0.94±0.30 AU vs. 1.06±0.34 AU, p = 0.20 in CHOW-DGAT1 vs. CHOW-control leg, **figure 5B**). Western blotting of UCP3 revealed a marked ∼33% decrease in protein content after overexpression of DGAT1 in HFD-fed rats, which was nearly significant (p = 0.09, HFD-DGAT1 vs. HFD-control leg, **figure 5C**). UCP3 protein levels were not significantly different between the HFD-DGAT1 overexpressing leg and both the CHOW-DGAT1 and CHOW-control legs (p = 0.88, CHOW-DGAT1 vs. CHOW-control leg, **figure 5C**).

### Electron microscopy, OXPAT

**Figure 6:**
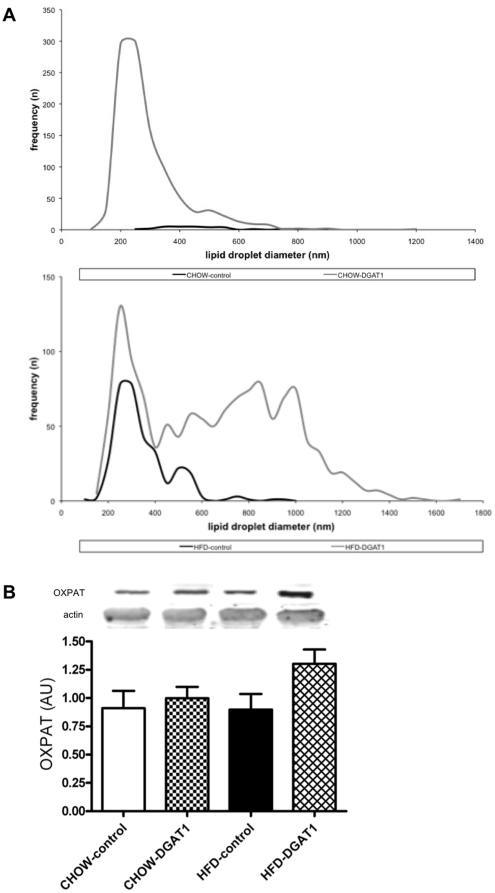
DGAT1 overexpression results in bigger lipid droplets in HFD-fed rats. (**A**) Electron microscopy revealed an increase in lipid droplet diameter in DGAT1 overexpressing TA muscle of HFD-fed rats. (**B**) Western blot analysis of OXPAT in rat TA muscle. Data are expressed as mean ± SEM (n = 10–12).

Oil Red O staining showed a marked increase in the number of intramyocellular lipid droplets upon DGAT1 overexpression, both in CHOW- and HFD-fed animals. However, electron microscopy revealed that DGAT1 overexpression on CHOW resulted in a massive increase in the number of lipid droplets of submicron size, which do not exceed the detection limit of light microscopy (**figure 6A** **, upper panel**). Upon high-fat feeding however, DGAT1 overexpression mainly increased the size of the lipid droplets, rather than the number (**figure 6A** **lower panel**). In contrast, in CHOW-fed rats, DGAT1 overexpression did not further increase the diameter of the lipid droplets (**Figure 6A** **, upper panel**). The increased size of the lipid droplets in the HFD-DGAT overexpressing leg, compared to the empty-vector control leg, corresponded with an increase in the lipid droplet coating protein OXPAT (p = 0.08, HFD-DGAT1 vs. HFD-control leg, and p = 0.14, CHOW-DGAT1 vs. CHOW-control leg, **figure 6B**).

### ATGL, CGI58, ADRP

**Figure 7:**
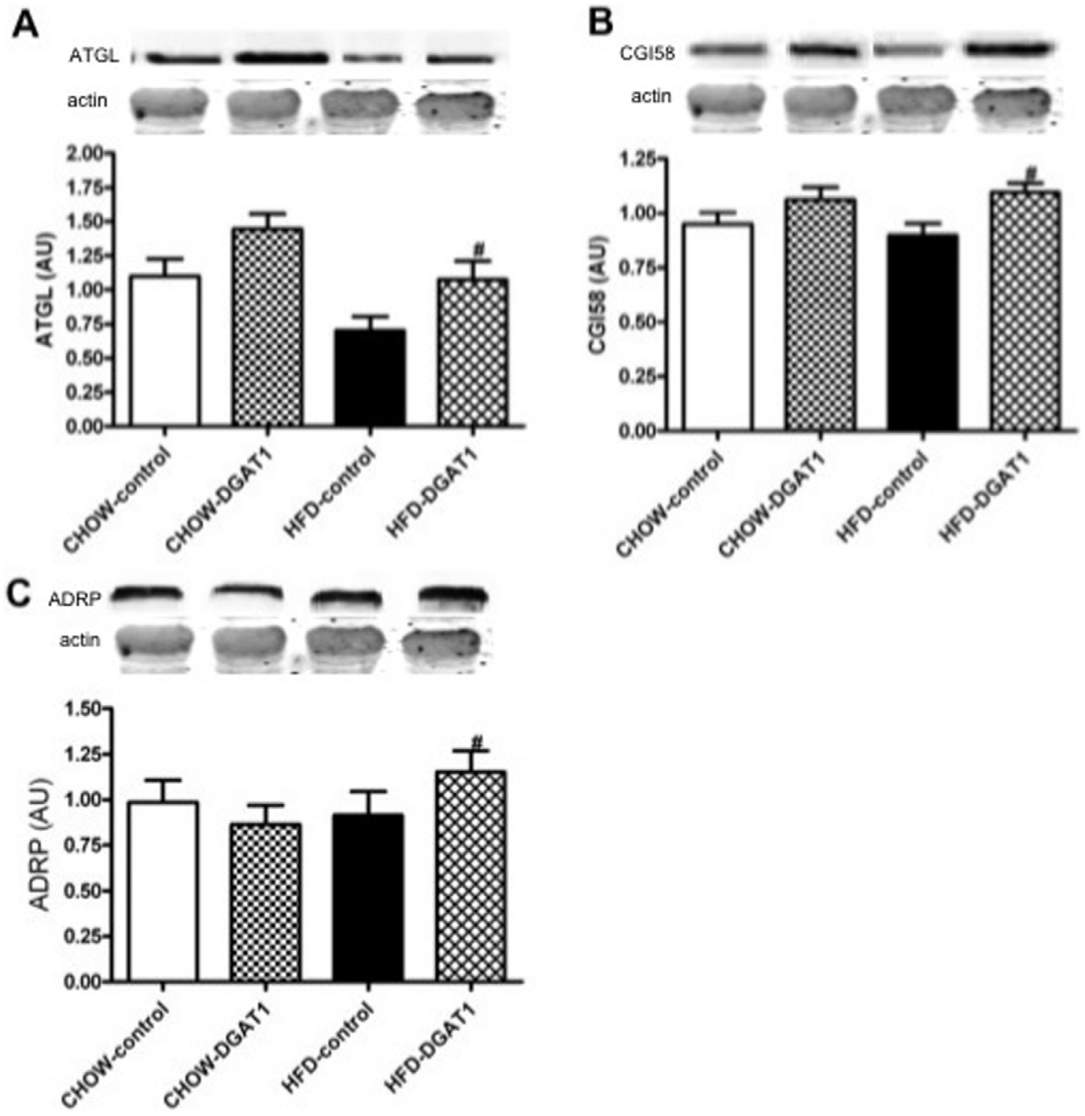
The turnover of DAG and TAG is increased in the DGAT1 overexpressing TA muscle. (**A**) Western blotting of ATGL, (**B**) CGI58 (**C**) and ADRP in rat TA muscle. Data are expressed as mean ± SEM (n = 10–12). **^#^** P<0.05 HFD-DGAT1 vs. HFD-control.

The finding that IMCL, DAG and markers of oxidative capacity were increased in DGAT1 overexpressing muscle may suggest that the turnover of IMCL is increased in the insulin-sensitive DGAT1 overexpressing legs. To test this hypothesis we determined the protein expression of ATGL and CGI58 as markers of intramyocellular triglyceride lipolysis. DGAT1 overexpression was associated with a ∼35% increase in ATGL content in the HFD-DGAT1 overexpressing leg compared to the control leg of the animals (p = 0.04, HFD-DGAT1 vs. HFD-control leg, **figure 7A**). DGAT1 overexpression in CHOW-fed rats tended to further enhance the ATGL protein content (p = 0.06, CHOW-DGAT1 vs. CHOW-control leg, **figure 7A**). Western blotting of CGI58, a cofactor of ATGL showed similar results. DGAT1 overexpression led to a ∼25% increase in CGI58 protein in the HFD-DGAT1 overexpressing leg compared to the control leg of the animals (p = 0.009, HFD-DGAT1 vs. HFD-control leg, **figure 7B**). DGAT1 overexpression in CHOW animals did not affect CGI58 protein expression (p = 0.16, CHOW-DGAT1 vs. CHOW-control leg, **figure 7B**). Accordingly, ADRP, a lipid droplet coating protein suggested to be involved in the regulation of lipolysis, was ∼25% increased in the HFD-DGAT1 overexpressing leg compared to the control leg of the animals (p = 0.05, HFD-DGAT1 vs. HFD-control leg, **figure 7C**). DGAT1 overexpression in CHOW animals had no effect on ADRP expression (p = 0.90, CHOW-DGAT1 vs. CHOW-control leg, **figure 7C**).

## Discussion

Elevated IMCL levels have been associated with the development of insulin resistance. Yet, insulin-sensitive, endurance trained athletes are also characterized by high IMCL levels. It has therefore been suggested that not IMCL, but rather intermediates of intramuscular triglyceride metabolism, such as DAG are causally related to insulin resistance. DGAT1 converts DAG into TAG in skeletal muscle, and increased DGAT1 activity has been suggested to explain the athlete's paradox [14], [16], [17]. In this study we show that DGAT1 overexpression in muscle of adult male Wistar rats indeed augments the storage of IMCL, but remarkably also results in increased skeletal muscle DAG content, even though a marked improvement in insulin sensitivity was observed. Our results suggest that increased intramuscular DAG and TAG turnover may underlie the beneficial effects on muscle insulin sensitivity.

In our study, short-term overexpression of DGAT1 resulted in a significantly higher intramyocellular lipid content both after CHOW and HFD, as expected based upon the primary function of DGAT1. Interestingly, electron microscopy revealed that overexpression of DGAT1 in animals receiving the HFD mainly resulted in an increased size of the lipid droplets, rather than an increased number. The increase in lipid droplet size in the HFD-DGAT1 overexpressing leg, compared with the empty vector control leg, was associated with an increase in the content of the lipid droplet coating proteins OXPAT and ADRP, which are thought to play a role in lipid synthesis and breakdown [28]. In CHO cells it was shown that ADRP is ubiquitinated and degraded by proteolysis when TAG levels decreased whereas inhibition of ADRP proteolysis led to a 2.8 fold increase in TAG levels [29]. Furthermore, improvement of human skeletal muscle insulin sensitivity is related to the upregulation of ADRP levels [30]. These data suggest that hydrolysis of TAG in lipid droplets might indeed be under control of ADRP. Also, OXPAT seems to promote long-chain fatty acid oxidation besides being involved in fatty acid-induced triacylglycerol accumulation [31].

By converting fatty acyl-CoA and DAG into TAG, DGAT1 is considered to modulate the levels of the lipotoxic fatty acid derivate DAG, which is marked as a possible causative fatty acid intermediate in the onset of insulin resistance [17]. Paradoxically, to our knowledge, this study is the first to show that myocellular DAG levels were increased upon muscle specific DGAT1 overexpression. The present study showed an increase in total DAG content upon DGAT1 overexpression in TA muscle compared to the empty-vector control leg, which was most pronounced upon high-fat feeding. In line with our observation, it has recently been shown by others that muscle-specific overexpression of DGAT1 resulted in a profound increase in IMCL [17]. In their model though, the increase in IMCL was associated with a reduction in muscle DAG content. Recently, the same group showed that muscle-specific transgenic overexpression of DGAT1 in mice enhanced muscle triglyceride synthesis as reflected by the 70% increase in muscle IMCL content; however, no data were reported on muscle DAG content [14]. In interpreting our results it is important to keep in mind that we used a specific muscle-orientated model to unilaterally overexpress DGAT1. Therefore, the effect of DGAT1 overexpression in muscle can be compared to control muscle in the same animal, preventing the influence of systemic differences, which are likely to interfere the comparison between a transgenic animal and a wild-type control. Indeed, the transgenic overexpression of DGAT1 by use of a muscle-specific MCK promotor also resulted in differences in body weight gain [14], [17]. The advantage of our model is that such whole-body phenotypic changes are prevented, allowing the conclusion that the results obtained in our study are truly muscle-specific.

In agreement with our hypothesis that increasing the lipogenic capacity of skeletal muscle can improve insulin sensitivity, the results of the present study indicate that DGAT1 overexpression did improve skeletal muscle insulin resistance. DGAT1 overexpression in left TA muscle of high-fat-fed rats was associated with a ∼16% higher uptake of bolus-injected 2DG in skeletal muscle compared to the sham-electroporated right leg of the animals. Previous studies confirm the maintenance of insulin sensitivity in skeletal muscle overexpressing DGAT1 by increasing the storage of fatty acids inside the muscle as TAG [14], [16], [17]. Moreover, these insulin-sensitizing effects observed in skeletal muscle overexpressing DGAT1 recapitulates the findings induced by exercise training, a phenomenon referred to as the athlete's paradox. Indeed, Liu et al. [17] compared the effects of upregulating myocellular DGAT1 expression with exercise and found that the mechanism underlying insulin sensitivity was similar in both conditions, namely the channelling of reactive fatty acid substrates into storage in the form of TAG. These results suggest that exercise-induced DGAT1 activation may explain the athlete's paradox. Here we confirm that DGAT1 overexpression may improve insulin sensitivity, but not via a decrease in total DAG content, but rather an elevated DAG content, although we can not exclude the possibility that a subfraction of “active” DAG was decreased in our study. Very interestingly, it was recently shown that endurance trained athletes are also not characterized by low, but rather by increased DAG levels in skeletal muscle [32], consistent with our observation of elevated DAG levels and improved insulin sensitivity. From these and our results, we suggested that increased turnover of DAG and TAG, especially upon high-fat feeding, may be beneficial for muscle insulin sensitivity. Accordingly, ATLG protein expression - the main triglyceride lipase in myotubes and in rat skeletal muscle [33] - was significantly elevated upon DGAT1 overexpression in left TA muscle of both HFD- and CHOW-fed rats compared to their control legs respectively. Moreover, the expression of CGI-58, an activating cofactor of ATGL [34], was significantly upregulated upon DGAT1 overexpression in rats receiving the HFD. Also, the lipid droplet coating protein ADRP, which is known to interact with CGI58 to regulate the access of this co-activator to the TAG stores, was significantly upregulated in the leg of HFD-fed rats subjected to DGAT1 overexpression [35], [36]. Hence, these data suggest that skeletal muscle triglyceride lipolysis may have been increased upon DGAT1 overexpression upon high-fat feeding. Again, this may resemble the endurance-trained state, as some [37] but not all endurance training studies [38], [39] observe an increase in ATGL content, indicating that the utilization of IMCL is increased.

In endurance training, increased muscle lipid content is balanced by elevated mitochondrial oxidative capacity [40], [41], allowing the oxidation of liberated fatty acids. Interestingly, in our overexpression model we show an increase in total OXPHOS protein content in the DGAT1 overexpressing TA muscle in rats receiving a HFD compared to their empty vector control legs. This pattern of protein expression was also shown for the individual complexes I, II and V of the OXPHOS complexes. In CHOW-fed animals, DGAT1 overexpression did not further enhance the expression level of the OXPHOS complexes. Also, PGC1α protein content, a marker of mitochondrial biogenesis, was significantly upregulated in the DGAT1 overexpressing leg of animals receiving a HFD, whereas no effect of DGAT1 overexpression on PGC1α protein levels were noticed upon CHOW-feeding. We have previously shown – as reviewed in [42] – that UCP3 protein content is elevated under conditions when fatty acid supply exceeds oxidative capacity, such as high-fat feeding and fasting, and UCP3 is reduced when oxidative capacity increases as is the case with endurance training. In line with our hypothesis, UCP3 was upregulated by HF feeding in the sham-electroporated leg, but DGAT1 overexpression reversed the increase in UCP3 protein content to levels observed in CHOW. This matches with the observed increase in the capacity of oxidative substrate degradation upon DGAT1 overexpression. These findings implicate that especially in case of increased delivery of free fatty acids (i.e. high fat feeding) to skeletal muscle, enhanced intramuscular lipid turnover together with the improvement in markers of mitochondrial function and biogenesis might be responsible for the improved skeletal muscle insulin sensitivity, even despite elevated muscle DAG levels.

Next to DAG, other fatty acid intermediates have been suggested to interfere with muscle insulin signalling. Thus, several studies have implicated a role for ceramides in the development of insulin resistance in conditions of increased fatty acid supply to skeletal muscle [43], [44], [45], [46]. Moreover, conditions of increased DGAT1 expression, such as exercise training [40], [47] have been linked to reductions in ceramide levels. Therefore, theoretically the insulin-sensitizing effect of DGAT1 overexpression could be due to reductions in ceramide levels. However, in our study, muscle ceramide content was not affected by increasing the intramyocellular lipid storage through DGAT1 overexpression, neither in animals on CHOW or on HFD. Thus, it seems that by specifically stimulating triglyceride biosynthesis in skeletal muscle, fatty acids are not directed towards *de novo* ceramide synthesis. More important, it shows that the positive effects of DGAT1 overexpression in muscle insulin sensitivity in our study could not be attributed to changes in ceramides levels. Alternatively, the formation of acylcarnitines, formed by incomplete β-oxidation, is also linked to insulin resistance in conditions of high fatty acid availability [48]. In our study, we observed that DGAT1 overexpression improved muscle insulin sensitivity in parallel with increased expression of markers of mitochondrial function such as PGC1α as well as the OXPHOS complexes. Liu et al. [14] also reported that DGAT1 overexpressing muscles appeared to have a greater mitochondrial fatty acid oxidation efficiency. These data could suggest that in our model acylcarnitines may be reduced, thereby explaining the improved insulin sensitivity. Unfortunately, due to the limited amount of muscle sample available and our prioritization of DAG and ceramide analysis, the reliable determination of long- and medium-chain acylcarnitines was not possible. However, we could reliably measure levels for free carnitine and acetylcarnitine, which were unaffected by our intervention. In general, increases in long-chain acylcarnitines are reflected in decreased free carnitine and acetylcarnitine levels [48]. The lack of effect of DGAT1 overexpression on free carnitine and acetylcarnitine levels therefore make it less likely that levels of acylcarnitines were changed in our current study.

Overall, we provide support for the hypothesis that in case of increased supply of fatty acids to skeletal muscle, as is the case with high-fat feeding, DGAT1 overexpression can improve insulin sensitivity. Interestingly, DGAT1 overexpression did not do so by lowering DAG, but more likely by increasing DAG and TAG turnover. Seemingly, by overexpressing DGAT1, a tight coupling between lipogenic and lipolytic pathways ensues and the capacity to oxidize fatty acids is upregulated in order to match the amount of fatty acids that are being offered. Apparently, only when there is a proper balance between the supply and oxidation of fatty acids in skeletal muscle, the lipid intermediate DAG may not exert harmful effects on insulin signalling.
